# Therapeutical and Pharmaceutical Notes

**Published:** 1900-11

**Authors:** W. J. Martin

**Affiliations:** Kankakee, Ill.


### THERAPEUTICAL AND PHARMACEUTICAL NOTES.

Under the Direction of W. J. Martin, M.D.C.,
KANKAKEE, ILL.

Antifebrine in Infectious Pneumonia. MM. Guillemain and Cadix have used this drug extensively in the treatment of the above-named disease. They gave ten drachms daily, divided into three to five doses, and came to the following conclusions:

1. The drug has a powerful antithermic action, as proved by the following cases:

Case I.—Morning temperature, third day, 40.2° C. Antifebrine administered three times. Evening temperature, 39.3° C.

Morning temperature, fourth day, 39.8°. Antifebrine administered three times. Evening temperature, 38.6°.

Morning temperature, fifth day, 39.2°. Antifebrine not administered. Evening temperature, 39.5°.

Case II.—Morning temperature, fourth day, 39.9°. Antifebrine administered three times. Evening temperature, 38.2°.

Morning temperature, fifth day, 39.1°. Antifebrine not administered. Evening temperature, 39°, instead of 38.2°, as it was the evening before at the same hour.

2. That the action of the drug is irregular and fleeting:

Case I.—Morning temperature, second day, 40.3°. Antifebrine administered at eleven, one, and three o'clock. Temperature, 4 p.m., 39.5°; at 6 p.m., 40.6°—a rise of 1.1° in two hours.

Morning temperature, third day, 40.8°. Antifebrine administered three times. Evening temperature, 40.8°.

Case II.—Morning temperature, third day, 39.9°. Antifebrine administered at seven, eight, nine, ten, and eleven o'clock. Temperature gradually declined 1.9° at mid-day, but at 6 p.m. again 40.4°.

From these cases it would appear that ten grammes is too little, and that, as it is rapidly eliminated, the drug should be administered frequently throughout the day.—*Veterinary Journal*, October, 1900.

Solutions of Formaldehyde. Dr. G. E. Crawford, in an article on "Formalin as an Antiseptic in General Surgery," gives the following formulæ: The 4 per cent. solution is made thus: Formalin, 5 oz.; sterilized water, 7½ pints. From this the following working solutions may be prepared: One-eighth per cent. solution of formalin or 1 in 2000 formaldehyde: 4 per cent. solution, ½ oz.; water, 15½ oz. One-quarter per cent. solution or 1 in 1000 formaldehyde: 4 per cent. solution, 1 oz.; water, 15 oz. Three-eighths per cent. solution, or 1 in 666 formaldehyde: 4 per cent. solution, 1½ oz.; water, 14½ oz. One-half per cent. solution, or 1 in 500 formaldehyde: 4 per cent. solution, 2 oz.; water, 14 oz. Five-eighths per cent. solution, or 1 in 400 formaldehyde: 4 per cent. solution, 2 oz.; water, 13½ oz. One per cent. solution, or 1 in 250 formaldehyde: 4 per cent. solution, 4 oz.; water, 12 oz.—*Can. Pharm. Journ.*

DISTINCT THERAPEUTIC PROPERTIES OF ALOES VARIETIES. A veterinary expert, in a contribution to the *Pharmaceutical Journal* on the uses of aloes, claims that while all the advances in modern therapy and pharmacy have been embraced by the scientific veterinarian, and aloin and the concentrated medicaments employed, the old bolus of Barbadoes aloes holds its own against all innovations. "It is a survival of the fittest; nothing else answers its purpose in the treatment of horses, although other varieties of aloes serve well enough in certain affections of cattle." As an aperient the Barbadoes variety acts with more certainty than any other, and without the accompaniment of abdominal pain, too often excited by Socotrine, Cape, or other aloes, an objection not even removed by the addition of warm aromatics, like ginger. Facts like these, even though they come from veterinary sources, should be borne in mind, now that there is a tendency to obliterate all lines of demarcation between the different varieties of aloes.—*Western Druggist.*

DIPHTHERIA ANTITOXIC GLOBULINS. Park (*Pediatrics*) says that investigations have shown that the antitoxic substances in the blood are closely combined with the globulins of the blood. Experiments made at the Research Laboratory of the New York Board of Health showed that injections of diphtheria toxin into horses increased the amount of globulin when the amount of antitoxin was increased. It was, therefore, decided to use this antitoxic globulin in cases of diphtheria. It was precipitated from the serum and an aqueous solution made, which was tried on forty-eight cases. Its injection was attended by rather more pain than the antitoxic serum. Its general effects were similar to those produced by the serum, the same rashes, etc., being produced. Therapeutic results were identical. On the whole, it was rather disappointing.—*Med. Stand.*

PREVENTION OF SURGICAL SHOCK. At the recent Medical Congress in Paris, Dr. F. B. Turck, of Chicago, in the section of general surgery, read a paper on "Prevention of Shock and Infection During Operation," which embodied reports of a series of experiments which show that the germs inoculated in animals when in shock proved pathogenic, but when not in shock they were non-pathogenic. Experiments proved that hot applications, made on animals when in shock, resuscitated, and no infection followed. The author is convinced that the application of hot-water bags in the cavity, added to the usual aseptic precautions, will prevent shock and lower mortality after operations.—*Ibid.*

FLUID EXTRACTS MADE WITH ACETIC ACID. There is a bare possibility that these extracts will be admitted into the next edition of the *United States Pharmacopœia.*

ANTHRAX TREATED WITH HYPODERMATIC INJECTIONS OF CARBOLIC ACID. Dr. W. E. Fisher, of Philadelphia, reports in the August number of the *Therapeutic Gazette* the successful treatment of a case of anthrax in man by the hypodermatic injection of carbolic acid. The patient, a male, was an employé of a large woollen mill in this city, the point of infection being a puncture of the forearm by a tack while at work. From a small pustule at the seat of injury cultures and cover-slip preparations were made, in which the presence of bacillus anthracis was demonstrated. The treatment consisted of injections of carbolic acid into and around the seat of the diseased area, as much as one drachm of the pure acid being injected in one day. This was continued until the patient had been given six drachms of the pure acid, and one drachm of a 10 per cent. solution of the acid also.

The results were most satisfactory, the patient making a complete recovery. At no time during the treatment was the carbolic acid detected in the urine, nor was there any noticeable pain or inflammation experienced by the patient from the acid.

ACTION OF THE THYROID GLAND. Steinler, Gautner, Kappler, and others have found by experiments performed upon animals from which the thyroid gland had been removed a decided retardation of the union of broken bones. This condition was readily overcome upon the administration to the animals of thyroid extract. These experiments demonstrate beyond a doubt that the thyroid gland exerts a decided physiological effect upon the nutrition of bone. This fact has been taken advantage of in the treatment of false joint in man by the internal administration of the thyroid extract.

The Swiss Pharmacopœia. At its recent session the permanent pharmacopœial commission of Switzerland decided to incorporate into the next edition of the *Pharmacopœia* seventy-four new articles, and to eliminate others to the extent of thirty-four. The text will not be in the Latin language. The use of fluid extracts for preparing tinctures, decoctions, and infusions will be declared not permissible.

Abortive Treatment of Boils. Dr. Jorissene, a French physician, aborts furuncles by applying an ointment consisting of one part of red mercuric oxide and ten parts of lanolin, rubbing it in for three or four minutes once a day or oftener in larger ones. Acne and whitlow are answerable to the same treatment.

Acetanilid in Epilepsy. Inglis (*Physician and Surgeon*) has obtained excellent results in the treatment of epilepsy by the use of the coal-tar products. He now uses acetanilid for this purpose, in doses of about five grains three times a day. This dose may in many cases be decreased if, after using it for some time, the patient remains free from the fits. He has found that the acetanilid treatment gives the best results in cases where the bromides have failed or have exhausted their beneficial effects. He, therefore, has come to the conclusion that where epilepsy is seen early it is best to put the patient first of all upon the bromide treatment. After this, if the drug loses its effect, the acetanilid may be given with reasonable hope of benefit. In canine and feline patients the above form of treatment will no doubt be found of value, more especially in those cases where the epilepsy is not due to the presence of intestinal parasites or structural alteration of the cranial walls, but arises from a functional neuroses of the central nervous system.—W. J. M.

